# Macrophage migration inhibitory factor (MIF) predicts survival in patients with clear cell renal cell carcinoma

**DOI:** 10.1002/2056-4538.12365

**Published:** 2024-03-04

**Authors:** Martyna Parol‐Kulczyk, Justyna Durślewicz, Laura Blonkowska, Radosław Wujec, Arkadiusz Gzil, Daria Piątkowska, Joanna Ligmanowska, Dariusz Grzanka

**Affiliations:** ^1^ Department of Clinical Pathomorphology, Faculty of Medicine, Collegium Medicum in Bydgoszcz Nicolaus Copernicus University Torun Poland; ^2^ Department of Pathophysiology, Faculty of Pharmacy, Collegium Medicum in Bydgoszcz Nicolaus Copernicus University Torun Poland

**Keywords:** EMT, epithelial–mesenchymal transition, MIF, macrophage migration inhibitory factor, ccRCC, clear cell renal cell carcinoma, β‐catenin, E‐cadherin, survival

## Abstract

Clear cell renal cell carcinoma (ccRCC) is one of the most common subtypes of renal cancer, with 30% of patients presenting with systemic disease at diagnosis. This aggressiveness is a consequence of the activation of epithelial–mesenchymal transition (EMT) caused by many different inducers or regulators, signaling cascades, epigenetic regulation, and the tumor environment. Alterations in EMT‐related genes and transcription factors are associated with poor prognosis in ccRCC. EMT‐related factors suppress E‐cadherin expression and are associated with tumor progression, local invasion, and metastasis. The aim of this study was to investigate the expression levels and prognostic significance of macrophage migration inhibitory factor (MIF), β‐catenin, and E‐cadherin in ccRCC patients. We examined these proteins immunohistochemically in tumor areas and adjacent normal tissues resected from patients with ccRCC. Analysis of the cancer genome atlas (TCGA) cohort was performed to verify our results. Kaplan–Meier analysis showed that median overall survival (OS) was significantly shorter in patients with tumors exhibiting high MIF^n^ and MIF^m‐c^ levels compared to those with low MIF^n^ and MIF^m‐c^ levels (*p* = 0.03 and *p* = 0.007, respectively). In the TCGA cohort, there was a significant correlation between MIF expression and OS (*p* < 0.0001). In conclusion, this study provides further evidence for the biological and prognostic value of MIF in the context of EMT as a potential early prognostic marker for advanced‐stage ccRCC.

## Introduction

Clear cell renal cell carcinoma (ccRCC) is one of the most common solid tumors, with approximately 81,800 new cases diagnosed each year in the United States [1]. About 30% of patients with ccRCC already have metastatic disease at the time of diagnosis, while another 30% are predisposed to metastases later in the course of their disease [2]. The remaining 40% are diagnosed with disease at the local stage. These data highlight the need to better understand the mechanisms by which metastasis occurs and to investigate critical points, such as the epithelial–mesenchymal transition (EMT) process, in order to develop appropriate targeted therapies.

The aggressiveness of cancer arises from its ability to detach from the primary site, intravasate and then extravasate cancer cells through the bloodstream, and adapt to new conditions in the surrounding microenvironment to form metastases. This aggressiveness is driven by the EMT process, which can be influenced by various inducers or regulators. Multiple studies have suggested that 5–10% of cancer cells in mouse model experiments exhibit an EMT program [3]. EMT is a program by which cells lose their epithelial appearance to gain mesenchymal characteristics. It is a physiological process that occurs during embryogenesis, but cancer cells can hijack this process to spread throughout the organism and form secondary tumors.

One potential EMT regulator that may provide an opportunity to target key pathways in ccRCC progression is the macrophage migration inhibitory factor (MIF). MIF is a proinflammatory cytokine with a regulatory role, in the innate and adaptive immune systems. Previous studies by Simpson *et al* showed that MIF indirectly affects tumor cells [4]. However, several studies have suggested that MIF may act directly on tumor cells as a proinflammatory cytokine, independent of its primary role [5]. Therefore, some researchers have focused on the role of MIF both as a mediator of the inflammatory process and as a factor promoting tumorigenesis in genitourinary cancers [6]. Some of the completed studies have shown that targeting MIF signaling could prevent the development and progression of many types of cancer [7]. The overexpression of MIF present in tumors not only confers an advantage in tumor growth but also confers a particular aggressiveness to tumor cells, allowing them to migrate and metastasize. The relationship between the MIF factor and tumorigenesis has been confirmed in various malignancies [6, 8]. The level of MIF expression in most cancers correlates with tumor progression and the ability of cancer cells to spread [9]. MIF can interact not only with tumor cells, but also with hematopoietic progenitor cells, mesenchymal stem cells, myeloid‐derived suppressor cells, and the tyrosine kinase receptor c‐mesenchymal–epithelial transition (c‐MET) factor to form the tumor microenvironment [10]. Physiologically, MIF circulates in blood serum, with an additional fraction secreted in the anterior pituitary gland, which activates monocytes/macrophages under the influence of specific factors [11].

Targeting MIF in cancer cells with the potential to spread through the body and metastasize to new secondary sites could then be transferred into clinical practice as a therapeutic option. Overall, it remains an interesting area of basic, translational, and clinical research to unravel the functions and complexity of MIF signaling related to the EMT process. In this paper, we aim to elucidate the correlations between MIF and clinicopathological features and underscore its significance as a regulator of the EMT process of patients diagnosed with ccRCC.

## Materials and methods

### Patients and tissue material

This study was performed on formalin‐fixed paraffin‐embedded tissue specimens from patients with ccRCC who underwent surgery at the General and Oncological Urology Clinic, Antoni Jurasz University Hospital No. 1 in Bydgoszcz (Poland). All patients had surgical resection of their ccRCC at the primary tumor site with a pretreatment biopsy approach. The evaluation and classification of resected tumors according to the American Joint Committee on Cancer eighth edition cancer staging system [12] were performed by two independent pathologists (RW and AG) using hematoxylin–eosin‐stained sections. The following patient clinical and pathological data were collected from the electronic medical records or histopathological diagnoses when available: age, histologic type, grade, lymph node status, distant metastasis status, or treatment information. Part of the same cohorts of patients and tissue samples were included in our previous article [13]. The study group included 99 patients (31 female, 68 male) in this research. The study protocol was approved by the Bioethics Committee at Collegium Medicum in Bydgoszcz of Nicolaus Copernicus University in Torun (no. 253/2018).

### Tissue macroarrays

Tissue macroarrays were constructed as previously described. In brief, representative tumor areas were used, and these were consolidated into one recipient block containing five distinct, large tissue fragments. Subsequently, tissue macroarray blocks were cut into 4‐μm thick sections using a manual rotary microtome (Accu‐Cut, Sakura Finetek, Torrance, CA, USA), placed on highly adhesive glass slides (Thermo Scientific, Menzel Gläser, SuperFrost® Plus, Braunschweig, Germany), and dried at 60 °C for 1 h.

### Immunohistochemical staining

Standardization, optimization, and selection of positive control sections for immunohistochemical staining were performed according to the instructions provided by the antibody manufacturers, and the data are available in the Human Protein Atlas (https://www.proteinatlas.org). The slides were subjected to immunohistochemical staining according to the previously described protocol [14, 15] using an automated system BenchMark® Ultra (Ventana Medical Systems, Tucson, AZ, USA). The primary antibodies used to evaluate the expression of MIF, β‐catenin, and E‐cadherin proteins were rabbit polyclonal anti‐MIF (HPA003868; 1:2,500; 32 min, LOT: 000010032, Sigma‐Aldrich, St. Louis, MO, USA), mouse monoclonal anti‐β‐catenin antibody (Ref: 760‐4242, pre‐diluted, 32 min, Ventana Medical Systems), and E‐cadherin mouse monoclonal antibody (Ref: 790‐4497, pre‐diluted, 24 min, Roche Diagnostics/Ventana). Primary antibodies were visualized using the ultraView‐Universal DAB Detection Kit (Roche Diagnostics/Ventana). Tissue sections were counterstained with hematoxylin (Roche Diagnostics/Ventana) for 12 min for MIF and 8 min for β‐catenin and E‐cadherin, followed by blue reagent (4 min, Roche Diagnostics/Ventana). Stained slides were dehydrated in ascending graded ethanol (80%, 90%, 96%, 99.8%), cleared in a series of xylenes (from I to IV), and finally coverslipped in Epredia mounting medium.

### Expression analysis

The stained slides were evaluated by two independent pathologists (RW and AG) using an Olympus BX53 (Olympus, Tokyo, Japan) at ×20 and ×40 magnification, with the assistance of a third pathologist (DG) in some cases. The pathologists were blinded to the clinical data of the patients. Immunostaining was assessed using the immunoreactivity scoring system [16], which is calculated by multiplying two factors: the percentage of cells/areas showing positive staining (ranging from 0 to 4) and the staining intensity (ranging from 0 to 3). The ultimate score, which falls within the range of 0–12, was dichotomized into two categories: low expression and high expression. This categorization was determined by a specific discriminatory threshold set using the Evaluate Cutpoints software [17]. The threshold values for defining low and high expression levels of nuclear MIF (MIF^n^), membranous‐cytoplasmic MIF (MIF^m‐c^), β‐catenin, and E‐cadherin were as follows: <4 and ≥4, <3 and ≥3, <4 and ≥4, and <1 and ≥1, respectively.

### *In silico* analysis of TCGA data

The survival and gene expression data for the cohort of 475 ccRCC patients from the cancer genome atlas (TCGA) were sourced from the UCSC Xena Browser (http://xena.ucsc.edu/). The RNA sequencing data pertaining to *MIF*, *CTNNB1*, and *CDH1* underwent normalization using the DESeq2 normalization method. Subsequently, the data were categorized into two expression groups: low level and high level, based on the cutoff values provided by the Evaluate Cutpoints software. Specifically, the cutoff point values for defining high and low expression of *MIF*, *CTNNB1*, and *CDH1* were as follows: <14.33 and ≥14.33, <14.06 and ≥14.06, and <11.91 and ≥11.91, respectively.

### Statistical analysis

Statistical analyses were conducted using SPSS software version 29.0 (IBM Corporation, Armonk, NY, USA) and GraphPad Prism version 10.0 (GraphPad Software, San Diego, CA, USA). The normality of the data distribution was assessed using the Shapiro–Wilk test. As the data were not normally distributed, the Mann–Whitney *U*‐test was employed to compare continuous variables. For categorical variables, comparisons were made using either Fisher's exact test or the Chi‐squared test. Survival analysis was conducted using the Kaplan–Meier method, and differences were evaluated using the log‐rank test. Disease duration data were censored at the last recorded time points, specifically, the date of death from any cause or the date of the last follow‐up. The median follow‐up duration was calculated using the reverse Kaplan–Meier estimator. Univariate and multivariate survival analyses were carried out using the Cox proportional hazards regression model. Hazard ratios (HRs) and their corresponding 95% confidence intervals (CIs) were also calculated. Statistical significance was defined as a *p* value <0.05.

## Results

### Immunohistochemical detection of MIF, β‐catenin, and E‐cadherin in ccRCC and adjacent normal tissue and their clinicopathological correlations

**Figure 1:**
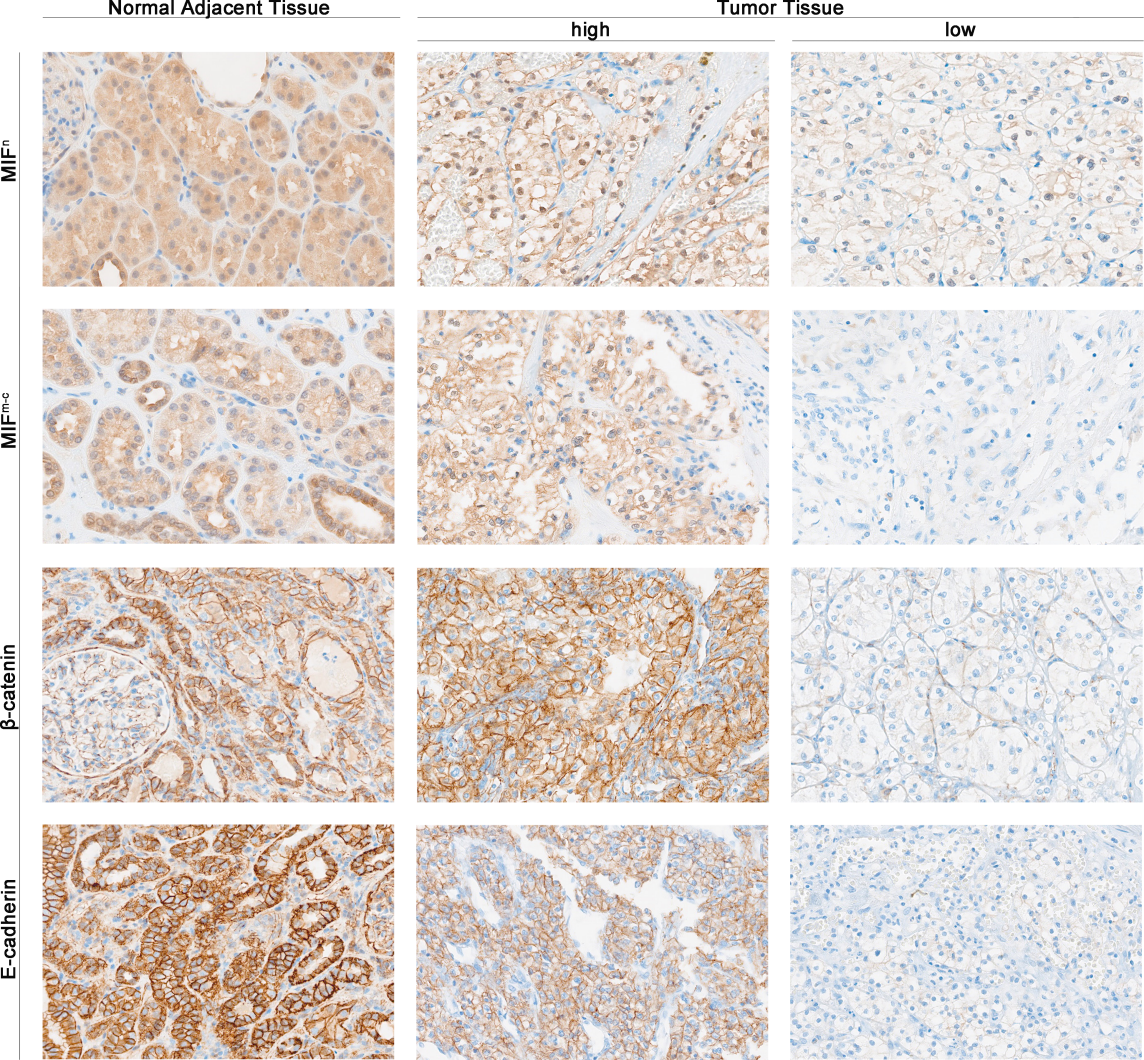
Representative images of immunohistochemical staining of normal adjacent tissue and tumor tissue from patients with ccRCC.

**Figure 2:**
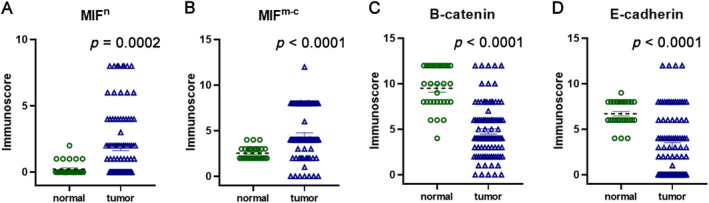
Comparison of the immunohistochemical expression of (A) nuclear MIF, (B) membranous‐cytoplasmic MIF, (C) β‐catenin, and (D) E‐cadherin in tumor and adjacent tissues of ccRCC patients.

The assessment of protein expression within our study cohort involved 99 cases of ccRCC and their corresponding nontumor adjacent tissues using immunohistochemistry. Immunohistochemical staining of MIF revealed its presence in the nuclear (MIF^n^) and membranous‐cytoplasmic (MIF^m‐c^) compartments of ccRCC cells (Figure 1). Comparison of MIF^n^ and MIF^m‐c^ expression levels between ccRCC tissues and adjacent nontumor tissues revealed a significant increase in ccRCC samples (for MIF^n^, *p* = 0.0002, and for MIF^m‐c^, *p* < 0.0001; Figure 2A,B). Using a predefined threshold, high MIF^n^ expression was identified in 24 cases (24.24%) of ccRCC tissues, while high MIF^m‐c^ expression was observed in 78 cases (78.79%). In the case of β‐catenin, the dominant expression pattern for tumor samples was membranous. As shown in Figure 1, compared to the adjacent tissue, tumor samples exhibited significantly decreased expression of β‐catenin (*p* < 0.0001; Figure 2C). Applying a cutoff from a predefined threshold, we identified high β‐catenin expression in 65 cases (65.66%) among ccRCC tissues. Immunohistochemical analysis of E‐cadherin revealed its membranous localization within ccRCC cells. Comparative assessment of E‐cadherin expression levels between ccRCC tissues and adjacent noncancerous counterparts unveiled a statistically significant downregulation in ccRCC specimens (*p* < 0.0001; Figure 2D). Employing a predetermined threshold, we identified high E‐cadherin expression in 57 cases (57.58%) among ccRCC tissues. Notably, the MIF^n^, MIF^m‐c^, and E‐cadherin expression profiles exhibited no discernible associations with the clinicopathological variables under investigation, as comprehensively outlined in Table 1. The expression of β‐catenin was associated with age (*p* = 0.0335).

**Table 1:** Association of MIF^n^, MIF^m‐c^, β‐catenin, and E‐cadherin protein expression in ccRCC with patient characteristics (*n* = 99)

		MIF^n^			MIF^m‐c^			β‐Catenin			E‐cadherin		
		+	−		+	−		+	−		+	−	
Clinicopathological feature	Number (%)	*n* = 24 (%)	*n* = 75 (%)	*p* value	*n* = 78 (%)	*n* = 21 (%)	*p* value	*n* = 65 (%)	*n* = 34 (%)	*p* value	*n* = 57 (%)	*n* = 42 (%)	*p* value
Gender													
Female	31 (31.3)	9 (9.1)	22 (22.2)	0.4592	26 (26.3)	5 (5)	0.5966	20 (20.2)	11 (11.1)	>0.9999	21 (21.2)	10 (10.1)	0.1934
Male	68 (68.7)	15 (15.2)	53 (53.5)		52 (52.5)	16 (16.2)		45 (45.5)	23 (23.2)		36 (36.4)	32 (32.3)	
Age													
≤65	58 (58.6)	12 (12.1)	46 (46.5)	0.3501	44 (44.3)	14 (14.1)	0.4611	33 (33.3)	25 (25.3)	**0.0335**	34 (34.3)	24 (24.3)	0.8386
>65	41 (41.4)	12 (12.1)	29 (29.3)		34 (34.3)	7 (7.1)		32 (32.3)	9 (9.1)		23 (23.2)	18 (18.2)	
Grade													
G1	25 (25.3)	10 (10.1)	15 (15.2)	0.0812	18 (18.2)	7 (7.1)	0.1356	16 (16.2)	9 (9.1)	0.6020	12 (12.1)	13 (13.1)	0.4371
G2	64 (64.6)	13 (13.1)	51 (51.5)		54 (54.5)	10 (10.1)		41 (41.4)	23 (23.2)		38 (38.4)	26 (26.3)	
G3	10 (10.1)	1 (1)	9 (9.1)		6 (6.1)	4 (4)		8 (8.1)	2 (2)		7 (7.1)	3 (3)	
pT status*													
T1	28 (28.3)	10 (10.1)	18 (18.2)	0.2483	22 (22.2)	6 (6.1)	0.4931	18 (18.2)	10 (10.1)	0.6261	17 (17.2)	11 (11.1)	0.9489
T2	28 (28.3)	5 (5.1)	23 (23.2)		20 (20.2)	8 (8.1)		17 (17.2)	11 (11.1)		16 (16.2)	12 (12.1)	
T3/T4	42 (42.4)	9 (9.1)	33 (33.3)		35 (35.3)	7 (7.1)		30 (30.3)	12 (12.1)		24 (24.3)	18 (18.2)	
pN status													
N0	92 (92.9)	23 (23.2)	69 (69.7)	>0.9999	72 (72.7)	20 (20.2)	>0.9999	58 (58.6)	34 (34.3)	0.0919	54 (54.5)	38 (38.5)	0.4532
N1	7 (7.1)	1 (1)	6 (6.1)		6 (6.1)	1 (1)		7 (7.1)	0 (0)		3 (3)	4 (4)	

Significant *p* values (*p* < 0.05) are shown in bold.

* Due to a lack of data in the medical record, the table does not include information on the pT status of one patient.

### Survival outcomes based on protein expression levels of MIF, β‐catenin, and E‐cadherin in ccRCC patients

**Figure 3:**
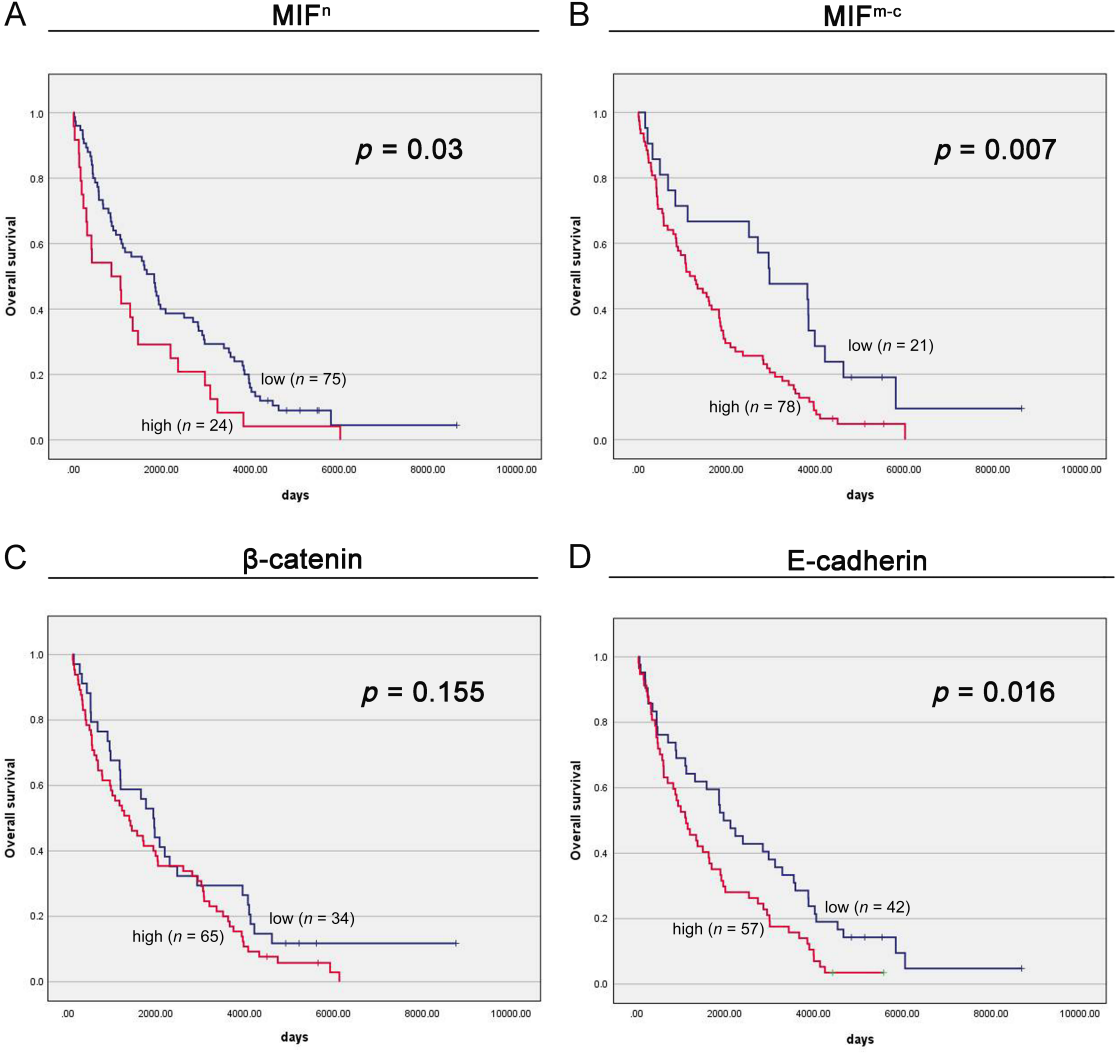
Kaplan–Meier survival curves stratified by (A) MIF^n^, (B) MIF^m‐c^, (C) β‐catenin, (D) E‐cadherin expression in ccRCC. Protein expression was measured using immunohistochemistry, and the calculated immunoreactivity scores were divided into two categories as specified in the Materials and methods section.

Kaplan–Meier analysis demonstrated that the median overall survival (OS) was significantly shorter in patients with tumors exhibiting high MIF^n^ levels compared to those with low MIF^n^ levels (859 days versus 1,824 days, *p* = 0.03; Figure 3A). Univariate Cox analysis revealed that high MIF^n^ protein expression predicted an unfavorable OS (HR: 1.68, 95% CI: 1.05–2.69; *p* = 0.03; Table 2). In the multivariate Cox proportional hazards model, MIF^n^ protein expression remained an independent prognostic factor for OS, even after adjusting for gender, age, grade, and cN status (HR: 1.98, 95% CI: 1.22–3.23; *p* = 0.01; Table 3). Furthermore, Kaplan–Meier survival analysis indicated that ccRCC patients with a high level of MIF^m‐c^ expression had lower OS rates than those with low MIF^m‐c^ expression levels (1,167 days versus 2,961 days, *p* = 0.007; Figure 3B). Univariate Cox analysis suggested that high MIF^m‐c^ protein expression predicted an adverse OS outcome (HR: 2.03, 95% CI: 1.2–3.43; *p* = 0.01; Table 2). In the multivariate Cox proportional hazards model, MIF^m‐c^ protein expression emerged as an independent prognostic factor for OS (HR: 2.66, 95% CI: 1.48–4.79; *p* < 0.01; Table 3). No association between β‐catenin expression and the survival of patients with ccRCC was demonstrated. Analysis of patient survival data showed that high E‐cadherin expression correlated with a lower OS rate than low expression (1,079 days versus 1,928 days, *p* = 0.016; Figure 3C). Univariate Cox analysis revealed that a high level of E‐cadherin predicted an unfavorable OS (HR: 1.68, 95% CI: 1.1–2.59; *p* = 0.02; Table 2). However, in the multivariate analysis, the result did not reach statistical significance (HR: 1.48, 95% CI: 0.95–2.3; *p* = 0.08; Table 3).

**Table 2:** Univariate analyses of prognostic factors in our cohort using the Cox proportional hazards model

	Univariate analysis			
Variable	HR	95% CI		*p* value
MIF^n^	1.68	1.05	2.69	0.03
MIF^m‐c^	2.03	1.20	3.43	0.01
β‐Catenin	1.37	0.89	2.13	0.16
E‐cadherin	1.68	1.10	2.59	0.02
Gender	0.57	0.37	0.89	0.01
Age	1.60	1.06	2.44	0.03
Grade	2.96	1.51	5.81	<0.01
pT status	3.80	1.69	8.55	0.53
N status	3.80	1.69	8.55	<0.01

**Table 3:** Multivariate analyses of prognostic factors in our cohort using the Cox proportional hazards model

	Multivariate analysis: MIF^n^				Multivariate analysis: MIF^m‐c^				Multivariate analysis: β‐catenin				Multivariate analysis: E‐cadherin			
Variable	HR	95.0% CI		*p* value	HR	95.0% CI		*p* value	HR	95.0% CI		*p* value	HR	95.0% CI		*p* value
MIF^n^	1.98	1.22	3.23	0.01	–	–	–	–	–	–	–	–	–	–	–	–
MIF^m‐c^	–	–	–	–	2.66	1.48	4.79	<0.01	–	–	–	–	–	–	–	–
β‐Catenin	–	–	–	–	–	–	–	–	1.19	0.75	1.91	0.46	–	–	–	–
E‐cadherin	–	–	–	–	–	–	–	–	–	–	–	–	1.48	0.95	2.30	0.08
Gender	0.59	0.38	0.92	0.02	0.66	0.42	1.03	0.07	0.58	0.37	0.90	0.02	0.65	0.41	1.03	0.06
Age	1.31	0.85	2.02	0.23	1.29	0.82	2.02	0.26	1.27	0.81	1.99	0.30	1.31	0.85	2.03	0.22
Grade	3.36	1.64	6.87	<0.01	5.03	2.28	11.11	<0.01	2.80	1.39	5.64	<0.01	2.78	1.38	5.61	<0.01
pT status	–	–	–	–	–	–	–	–	–	–	–	–	–	–	–	–
N status	4.02	1.76	9.21	<0.01	3.29	1.45	7.47	<0.01	3.29	1.43	7.57	<0.01	3.31	1.45	7.53	<0.01

Dash (–) indicates that the variable was not included in the multivariate analysis.

### Correlation between protein expression levels of MIF, β‐catenin, and E‐cadherin in ccRCC patients

A statistically significant, albeit weak, positive association was observed between the expression levels of MIF^n^ and MIF^m‐c^ (*r* = 0.2871, *p* = 0.004), MIF^m‐c^ and E‐cadherin (*r* = 0.2158, *p* = 0.032), and β‐catenin and E‐cadherin (*r* = 0.2638, *p* = 0.008).

### Associations between mRNA expression of *MIF* , *CTNNB1* , and *CDH1* and clinical features

**Figure 4:**
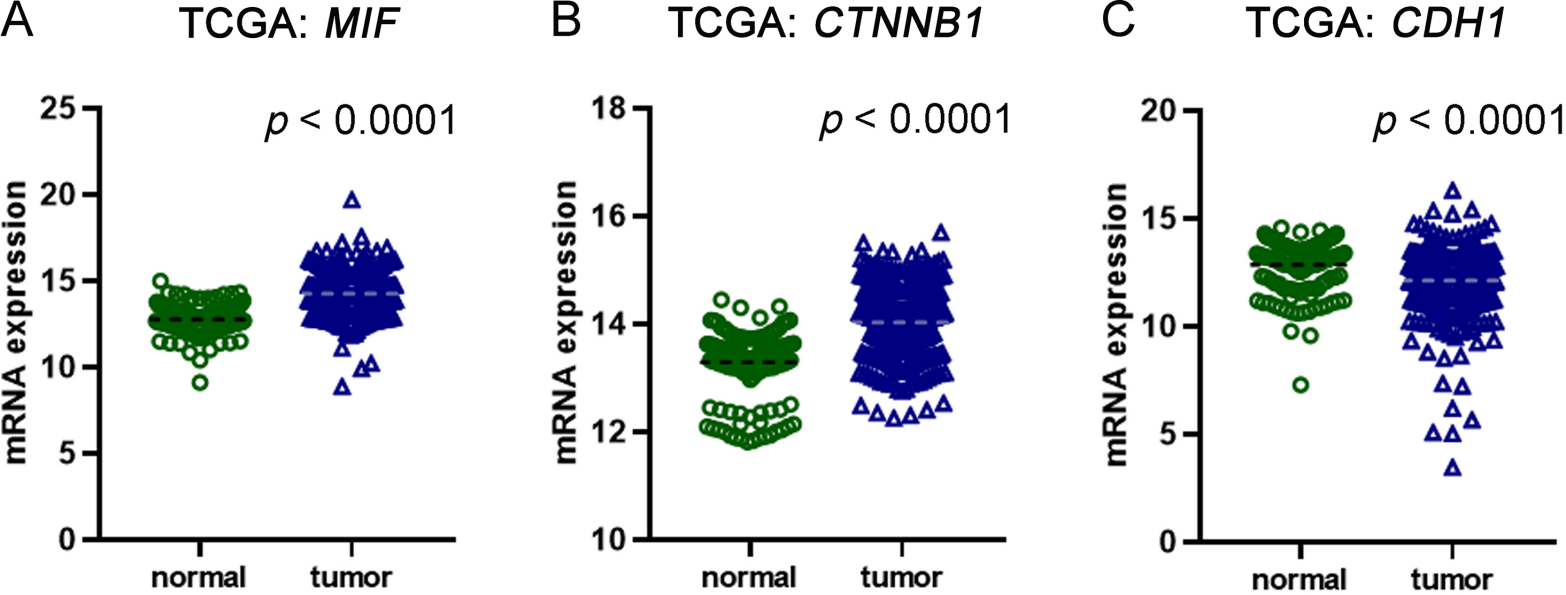
mRNA expression of *MIF*, *CDH1*, and *CTNNB1* in ccRCC.

Analysis of mRNA sequencing data unveiled a substantial increase in the expression levels of *MIF* and *CTNNB1* in ccRCC when compared to normal kidney tissues (Figure 4A,B). Conversely, *CDH1* expression levels exhibited a significant reduction in ccRCC in comparison to normal kidney tissues (Figure 4C). Following the determination of the optimal cutpoints utilizing the Evaluate Cutpoints software, we identified an upregulation of MIF in 210 (44.21%) cases of ccRCC, with concomitant downregulation observed in the remaining 265 (55.79%) cases. Moreover, our investigation unveiled an upregulation of *CTNNB1* in 242 (50.95%) ccRCC, with downregulation apparent in the remaining 233 (49.05%) cases. In addition, we found upregulation of *CDH1* in 321 (67.58%) ccRCC cases, alongside concurrent downregulation in the remaining 154 (32.42%) cases. Significant correlation was observed between *MIF* status and grade (*p* = 0.0472), pT status (*p* = 0.0002), and stage (*p* = 0.0005; Table 4). In addition, there was a notable trend indicating a potential correlation between *CTNNB1* levels and pT status (*p* = 0.0697) as well as pN status (*p* = 0.0661; Table 4), and a significant correlation was established between *CTNNB1* levels and stage (*p* = 0.0212). Furthermore, we found associations between *CDH1* expression levels and pT status (*p* = 0.0006) and stage (*p* = 0.0306; Table 4).

**Table 4:** Association of *MIF*, *CDH1*, and *CTNNB1* mRNA expression in ccRCC with patient characteristics (*n* = 475)

		MIF			CTNNB1			CDH1		
		+	−		+	−		+	−	
Clinicopathological feature	Number (%)	*n* = 210	*n* = 265	*p* value	*n* = 242	*n* = 233	*p* value	*n* = 321	*n* = 154	*p* value
Gender										
Female	163 (34.32)	67	96	0.3324	83	80	>0.9999	109	54	0.8368
Male	312 (65.68)	143	169		159	153		212	100	
Age										
≤60	239 (50.32)	113	141	>0.9999	123	116	0.8545	159	80	0.6257
>60	236 (49.68)	105	131		119	117		162	74	
Grade										
G1	11 (2.32)	3	8	**0.0472**	7	4	0.5348	7	4	0.542
G2	203 (42.74)	77	126		109	94		144	59	
G3	189 (39.79)	93	96		90	99		125	64	
G4	72 (15.16)	37	35		36	36		45	27	
pT status										
T1	237 (49.89)	82	155	**0.0002**	134	103	**0.0697**	166	71	**0.0006**
T2	61 (12.84)	36	25		31	30		45	16	
T3	167 (35.16)	88	79		72	95		109	58	
T4	10 (2.11)	4	6		5	5		1	9	
pN status										
Nx	235 (49.47)	111	124		121	114		160	75	>0.9999
N0	225 (47.37)	91	134	0.4183	117	108	**0.0661**	151	74	
N1	15 (3.16)	8	7		4	11		10	5	
Stage										
I	234 (49.26)	81	153	**0.0005**	133	101	**0.0212**	164	70	**0.0306**
II	50 (10.53)	28	22		27	23		37	13	
III	119 (25.05)	61	58		55	64		82	37	
IV	72 (15.16)	40	32		27	45		38	34	

Significant *p* values (*p* < 0.05) are shown in bold.

### Survival outcomes based on mRNA expression levels of *MIF* , *CTNNB1* , and *CDH1* in ccRCC patients

**Figure 5:**
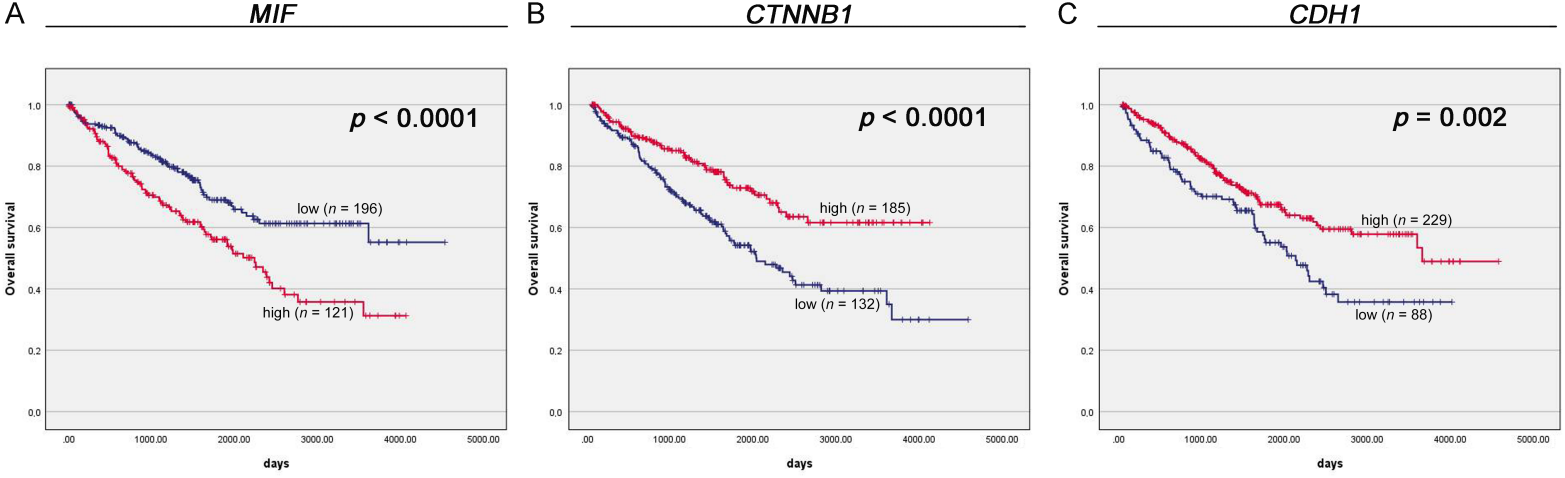
Kaplan–Meier survival curves stratified by (A) *MIF*, (B) *CTNNB1*, and (C) *CDH1* mRNA levels in TCGA cohort.

Survival analysis conducted on the TCGA cohort unveiled a significant correlation between *MIF* expression and OS (2,241 days versus undefined days, *p* < 0.0001; Figure 5A). In univariate Cox analysis, high *MIF* mRNA expression emerged as a robust predictor of unfavorable OS (HR: 1.79, 95% CI: 1.31–2.46; *p* < 0.01; Table 5). In the multivariate Cox proportional hazards model, *MIF* mRNA expression maintained its independent prognostic significance for OS, even after adjusting for factors such as grade and TNM stage (HR: 1.54, 95% CI: 1.12–2.12; *p* < 0.01; Table 6). Moreover, Kaplan–Meier survival analysis indicated that ccRCC patients with elevated mRNA levels of *CTNNB1* exhibited higher OS rates compared to those with lower expression levels (undefined days versus 1,986 days, *p* < 0.0001; Figure 5B). Univariate Cox analysis suggested that high *CTNNB1* mRNA expression was associated with a favorable OS outcome (HR: 0.51, 95% CI: 0.37–0.71; *p* < 0.01; Table 5). In the multivariate Cox proportional hazards model, *CTNNB1* mRNA expression emerged as an independent prognostic factor for OS (HR: 0.58, 95% CI: 0.42–0.81; *p* < 0.01; Table 6). Furthermore, the Kaplan–Meier survival analysis revealed that ccRCC patients with higher mRNA levels of *CDH1* experienced significantly extended OS compared to those with lower expression levels (3,615 days versus 2,090 days, *p* = 0.002; Figure 5B). Univariate Cox analysis indicated that elevated *CDH1* mRNA expression was associated with a favorable OS outcome (HR: 0.61, 95% CI: 0.45–0.84; *p* < 0.01; Table 5). In the multivariate Cox proportional hazards model, *CDH1* mRNA expression continued to be an independent prognostic factor for OS (HR: 0.61, 95% CI: 0.44–0.84; *p* < 0.01; Table 6).

**Table 5:** Univariate analyses of prognostic factors in the TCGA cohort using the Cox proportional hazards model

	Univariate analysis			
Variable	HR	95% CI		*p* value
*MIF*	1.79	1.31	2.46	<0.01
*CTNNB1*	0.51	0.37	0.71	<0.01
*CDH1*	0.61	0.45	0.84	<0.01
Gender	0.95	0.68	1.31	0.74
Age	1.01	1.00	1.02	0.08
Grade	1.36	0.98	1.87	0.06
pT status	3.19	2.31	4.39	<0.01
N status	3.65	1.93	6.89	<0.01
TNM stage	3.61	2.59	5.02	<0.01

**Table 6:** Multivariate analyses of prognostic factors in the TCGA cohort using the Cox proportional hazards model

	Multivariate analysis: *MIF*				Multivariate analysis: *CTNNB1*				Multivariate analysis: *CDH1*			
Variable	HR	95% CI		*p* value	HR	95% CI		*p* value	HR	95% CI		*p* value
*MIF*	1.54	1.12	2.12	0.01	–	–	–	–	–	–	–	–
*CTNNB1*	–	–	–	–	0.58	0.42	0.81	<0.01	–	–	–	–
*CDH1*	–	–	–	–	–	–	–	–	0.61	0.44	0.84	<0.01
Grade	1.13	0.82	1.57	0.46	1.16	0.84	1.61	0.36	1.13	0.82	1.57	0.46
TNM stage	3.40	2.43	4.74	<0.01	3.36	2.41	4.70	<0.01	3.59	2.57	5.00	<0.01

### Correlation between mRNA expression levels of *MIF* , *CTNNB1* , and *CDH1* in ccRCC patients

A statistically significant weak negative association was identified between the expression levels of *MIF* and *CTNNB1* (*r* = −0.1453, *p* = 0.001). There were no other statistically significant correlations observed.

## Discussion

The role of MIF, one of the key factors stimulating cancer progression, is currently unknown related to the EMT in ccRCC. Therefore, the alterations of MIF, β‐catenin, and E‐cadherin were investigated in relation to clinicopathology and OS. To elucidate the correlation between MIF, β‐catenin, and E‐cadherin expression and invasion or metastasis in ccRCC, the immunohistochemical status of tissue proteins was examined and an *in silico* study of these mRNAs in ccRCC patients based on the TCGA dataset was carried out.

It is well known that *MIF* is a gene induced by hypoxia through the regulation of hypoxia‐inducible factor‐1 (HIF‐1). MIF functions as a direct transcriptional target of HIF [18]. In the study by Oda *et al*, MIF was found to be regulated by HIF‐1 activity in a p53 signaling pathway. MIF is involved in fundamental processes including cell proliferation and survival, angiogenesis, and tumor invasiveness, in addition to its potent effects on the immune system. This cooperation between MIF and HIF‐1α protein stabilization and transactivation activity suggests a mechanism of tumor progression by MIF [19]. Another study confirms the upregulation of MIF levels during hypoxic and hypoglycemic conditions and suggests that MIF factor may play a key role in neovascularization in patients with glial tumors [20]. Du *et al*, in their study of ccRCC, identified MIF as a factor involved in protumorigenic signaling that acts in an autocrine manner to promote cancer. They found MIF to be a minimally invasive and useful marker of cancer stage [21]. Baugh *et al* proposed a model in which hypoxia‐activated MIF is regulated by HIF‐1 and amplified by degradation of CREB [22].

MIF's promotion of proliferation and neuronal differentiation of neural stem/progenitor cells through the Wnt/β‐catenin signaling pathway was confirmed by Zhang *et al* in 2013 [23]. To date, a functional interaction between MIF and the Wnt/β‐catenin pathway has not been described in any malignancy. However, both MIF and β‐catenin proteins have been found to be involved in the EMT process in various tumors. Protein co‐expression between MIF and β‐catenin levels in prostate cancer was measured in the study conducted by Parol‐Kulczyk *et al* [14]. Spearman rank coefficient revealed a weak negative correlation between total β‐catenin expression and nuclear expression of MIF protein. This may indicate the involvement of both factors in tumor progression and initiation of the EMT to metastasis cascade. Similarly, the TCGA analysis of the correlation between levels of *MIF* gene expression in ccRCC patients revealed significant association with *CTNNB1* expression at low level (*r* = −0.145, *p* = 0.001). Furthermore, Yang *et al* reported that cells with increased expression of MIF led to loss of E‐cadherin and increased levels of N‐cadherin, vimentin, and Zeb‐1 in pancreatic cancer [24]. The switch from E‐cadherin to N‐cadherin is considered as a hallmark of EMT, where E‐cadherin maintains the integrity of the epithelial architecture, whereas N‐cadherin stimulates metastasis [25]. In our study, there is no clear relationship between MIF and E‐cadherin levels; however, multivariate analysis of *in silico* TCGA data shows that both membranous‐cytoplasmic and nuclear MIF, and E‐cadherin correlate with N status in ccRCC patients, which may confirm somehow the involvement of these factors in the induction of metastasis.

Increased ccRCC aggressiveness and the possibility to initiate the EMT process were mainly reflected by altered MIF expression. The most common expression pattern of MIF in ccRCC tissues was membranous‐cytoplasmic expression, which was not found to correlate with clinicopathological features in our cohort. We also observed nuclear staining of MIF factor in cancer cells without any significant correlations with clinicopathology. However, the Kaplan–Meier analysis showed that the median OS was significantly shorter in patients with tumors that expressed high levels of MIF^n^ and MIF^m‐c^ compared to patients with tumors that expressed low levels of MIF^n^ or MIF^m‐c^.

The pro‐metastatic role of MIF factor has been reported in different studies. Yang *et al* have shown that MIF may influence a tumor suppressor gene named *NR3C2* encoding a mineralocorticoid receptor and through reducing the NR3C2 levels may stimulate the growth, migration, and invasion of cancer cells [24]. In turn, Huang *et al* found that inhibiting MIF expression with short hairpin RNA led to the termination of EMT by activating the reverse process, MET [26]. Similarly, reduction of MIF levels markedly suppressed EMT in a salivary adenoid cystic carcinoma cell line [27]. Therefore, the studies performed to date suggest a strong link between the MIF molecule and the induction of EMT.

Our data strongly emphasize that both fractions of MIF factor in cancer cells correlate with worse survival in patients with ccRCC. Kaplan–Meier survival analysis showed that ccRCC patients with high expression of MIF^m‐c^ or MIF^n^ were at risk of shorter survival in ccRCC. It appears that patients with high expression of MIF^m‐c^ or MIF^n^ protein have no chance of surviving more than 2 or 3 years, respectively, from the time of cancer diagnosis. Recent studies have identified MIF as a biomarker predictive of poor prognosis in glioma, triple negative breast cancer, or head and neck squamous cell carcinoma [28, 29, 30]. Interestingly, in human glioblastoma cells, elevated MIF fractions were found to increase the levels of mesenchymal markers. In addition, the enhancement of EMT was investigated *in vivo*, where treatment with recombinant human MIF resulted in an increase in tumor size and EMT. This effect was inhibited by blocking the CXCR4‐AKT pathway [31]. In our study, we demonstrated that MIF, E‐cadherin, and β‐catenin were each associated with indicators such as pT and stage. In patients with high‐grade osteosarcoma, Han *et al* found that MIF overexpression was associated with poor OS and metastasis‐free survival [32]. In two other studies on patients with esophageal squamous cell carcinoma and lung squamous cell carcinoma, high MIF levels compared to low MIF levels were associated with worse OS or disease‐specific survival and disease‐free survival, respectively [23, 33], supporting our study results. Some studies in pancreatic ductal adenocarcinoma and gastric cancer showed that elevated expression of MIF corresponded with unfavorable OS [34, 35]. In addition, Kang *et al* demonstrated an association between MIF levels and OS as well as recurrence‐free survival in oral squamous cell carcinoma [36]. These findings may suggest that the tissue status of MIF^n^ and MIF^m‐c^ is an important prognostic indicator in ccRCC. The high likelihood of increased EMT and metastasis may be related to the high malignant potential of ccRCC tumors with elevated MIF expression.

E‐cadherin, as a key member of the intracellular adhesion molecule family, plays a critical role in cell adhesion and differentiation and is highly expressed by epithelial cells [37]. Recently, the downregulation of E‐cadherin has been shown to be a prognostic factor at the time of hepatocellular carcinoma diagnosis [38, 39, 40]. Using Kaplan–Meier analysis, our study showed a clear trend in the survival of ccRCC patients in relation to the expression level of E‐cadherin protein. In fact, we found that higher E‐cadherin expression in cancer cells was associated with poor survival in ccRCC patients. However, this association was not confirmed by multivariate analysis. The result of our study was not supported by *in silico* analysis, which showed that high *CDH1* gene expression correlated with favorable survival in patients with ccRCC. This finding is consistent with a landmark study by Yonemura *et al* using 98 primary gastric cancer specimens and anti‐E‐cadherin monoclonal antibody. They observed typical homogeneous expression of E‐cadherin located in the cell membrane of normal epithelial cells of gastric tissues whereas, in approximately 71% of gastric cancer tissues, the cancer cells with decreased and heterogeneous expression of E‐cadherin tended to infiltrate the gastric wall and invade the adjacent lymph nodes or peritoneal surface. Therefore, decreased E‐cadherin levels were associated with shorter survival (*z* = 3.98, *p* = 0.00086) [41].

Cancer aggressiveness and resistance to therapy are characteristics attributed to the initiation of EMT by cancer cells. Identification of key signaling pathways involved in EMT activation may provide better treatment options for ccRCC patients in the future. The signaling pathways that activate the EMT process in cancer cells are complex and involve many inducers, regulators, and effectors of this process, often linking inflammation to cancer and the EMT event. Cancer cells may acquire invasive properties at an earlier stage of tumor development, which is promoted by EMT in the cancer microenvironment through the activation of inflammatory inducers such as MIF. One study has shown that MIF‐induced EMT in pancreatic cancer cells utilizes the miR‐200/ZEB/E‐cadherin axis, providing a potential strategy for targeting MIF to inhibit the miR‐200/ZEB interaction and consequently EMT [42]. This may represent a significant development in treating advanced RCC patients and will potentially lead to new treatment options for such patients in the future.

In conclusion, the hypothesis that MIF as an EMT inducer may promote tumor aggressiveness in ccRCC patients is supported by the results of our work. Currently, clinical trials are underway using the MIF molecule to treat patients with ccRCC. One completed Phase 1 study is Clinical Trial No. 391101 (EudraCT number: 2013‐002870‐31; https://clinicaltrials.gov/study/NCT01765790) to evaluate the safety, tolerability, pharmacokinetics, and pharmacodynamics of the MIF antibody in patients with solid malignancies and in patients with metastatic adenocarcinoma of the colon or rectum. We propose to consider the MIF molecule as a promising candidate in resected patients, which may improve the prognosis and survival of patients with ccRCC.

It should be acknowledged that our study has certain limitations related to the size of the examined cohort. This limitation emphasizes the need for caution in interpreting the results and underscores the importance of conducting further research with larger cohorts to confirm and strengthen our findings. In addition, the conducted analyses were supplemented with data obtained from TCGA, which provided valuable information but has certain limitations. A notable limitation of our study is the predominant representation of earlier stages of ccRCC in the TCGA dataset. The overrepresentation of these cases may limit the extrapolation of our survival analysis results to patients in more advanced stages of the disease. Future studies with a more diverse and balanced representation of different stages of ccRCC are warranted to verify and extend our observations.

## Author contributions statement

MPK conceived and designed the study. MPK and JD collected data. JD, RW, AG and MPK analyzed and interpreted results. MPK, JD and LB prepared the draft manuscript. LB, DP and JL provided technical support. DG supervised the work. All authors reviewed the results and approved the final version of the manuscript.

## Data Availability

The datasets generated and analyzed during the current study can be obtained from the corresponding author upon reasonable request.
